# Anchoring of a dye precursor on NiO(001) studied by non-contact atomic force microscopy

**DOI:** 10.3762/bjnano.9.26

**Published:** 2018-01-23

**Authors:** Sara Freund, Antoine Hinaut, Nathalie Marinakis, Edwin C Constable, Ernst Meyer, Catherine E Housecroft, Thilo Glatzel

**Affiliations:** 1 Department of Physics, University of Basel, Klingelbergstrasse 82, 4056 Basel, Switzerland; 2 Department of Chemistry, University of Basel, BPR 1096, Mattenstrasse 24a, 4058 Basel, Switzerland

**Keywords:** metal oxide, nickel(II) oxide (NiO), non-contact atomic force microscopy, p-type semiconductor, sub-molecular resolution

## Abstract

The properties of metal oxides, such as charge-transport mechanisms or optoelectronic characteristics, can be modified by functionalization with organic molecules. This kind of organic/inorganic surface is nowadays highly regarded, in particular, for the design of hybrid devices such as dye-sensitized solar cells. However, a key parameter for optimized interfaces is not only the choice of the compounds but also the properties of adsorption. Here, we investigated the deposition of an organic dye precursor molecule on a NiO(001) single crystal surface by means of non-contact atomic force microscopy at room temperature. Depending on the coverage, single molecules, groups of adsorbates with random or recognizable shapes, or islands of closely packed molecules were identified. Single molecules and self assemblies are resolved with submolecular resolution showing that they are lying flat on the surface in a trans-conformation. Within the limits of our Kelvin probe microscopy setup a charge transfer from NiO to the molecular layer of 0.3 electrons per molecules was observed only in the areas where the molecules are closed packed.

## Introduction

Inorganic substrates functionalized with organic molecules are nowadays highly regarded materials for emerging hybrid technologies including molecular electronics, photocatalysts or photovoltaics such as dye-sensitized solar cells (DSSCs) [1–4]. In the latter field, the wide band gap n-type semiconductor TiO_2_ has become one of the most common metal oxides for the design of classical n-type DSSCs, and is therefore a widely studied material, in particular in the field of scanning probe microscopy (SPM) [5]. Through the adsorption of a large variety of dye molecules, the ability of sensitized TiO_2_ to absorb light can be triggered and tuned. Thus, the possibility of designing photoactive devices with anodes consisting of nanostructured and functionalized TiO_2_ leads to numerous fundamental studies including those at the molecular or sub-molecular scale [6–14]. In contrast, the synthesis and characterization of p-type wide band gap metal oxide materials and especially the fabrication and analysis of p-type DSSCs with a photoactive cathode, are less commonly discussed [15–16]. Such p-type DSSCs are a first step towards the fabrication of hybrid tandem solar cells where both electrodes will be photoactive allowing for higher open-circuit voltages and energy conversion efficiencies [15,17].

The first reported p-type wide band gap metal oxide material was NiO [18], but other promising materials have also been intensively researched [19–21]. However, for the application in p-DSSCs, NiO is the most extensively studied material [22–24]. A detailed understanding of the adsorption properties of dye molecules on NiO cathodes is therefore of crucial importance for the comprehension and design of improved and more efficient devices.

NiO single-crystal surfaces functionalized with adsorbed dye molecules are ideal candidates to analyze the fundamental properties of such systems by SPM techniques. Because of the large band gap of NiO, which is reported to lie between 3.5 and 4.3 eV [25–29], and given that scanning tunnelling microscopy (STM) can only be performed on thin NiO films grown on metals [30–31], non-contact atomic force microscopy (nc-AFM) in ultra-high vacuum is the technique of choice. Due to its hardness and high reactivity, preparation of atomically clean NiO crystal surfaces is challenging and few studies, focusing mainly on magnetic properties, have been reported [32–40]. The adsorption of Co–salen, a paramagnetic complex, onto these surfaces was recently reported [41].

One of the possibilities of sensitizing NiO surfaces for an optimized photon absorption is an on-surface dye synthesis, which has earlier been termed the “surfaces-as-ligands” approach [42]. The first ligand is designed to anchor to the surface through groups such as carboxylic or phosphonic acids and has a metal-binding domain such as 2,2′-bipyridine (bpy). In a second step, the ligand-functionalized surface is either directly exposed to a homoleptic metal (typically copper) complex to undergo a ligand-exchange reaction to assemble a surface-bound heteroleptic dye, or is first capped with a metal ion followed by the ancillary ligand to complete the active dye [43–45].

**Figure 1:**
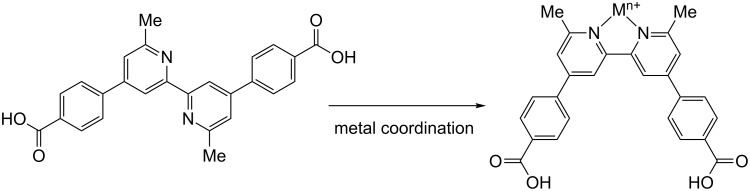
Structure of 4,4′-di(4-carboxyphenyl)-6,6′-dimethyl-2,2′-bipyridine. The trans-conformation of DCPDMbpy (left) changes to a *cis*-conformation upon coordination of a metal ion M*^n+^* (right).

We present in this paper, high-resolution structural and electrical measurements obtained by nc-AFM of a typical anchoring ligand (DCPDMbpy), based on a 6,6′-dimethyl-2,2′-bipyridine metal-binding domain with two 4-carboxyphenyl anchoring groups (see Figure 1), adsorbed on an atomically clean NiO(001) crystal surface. It adsorbs either as single molecule or forms specific assemblies increasing in size from small clusters up to complete islands inducing a clear change of the surface potential.

## Results and Discussion

### Atomic resolution of NiO(001)

**Figure 2:**
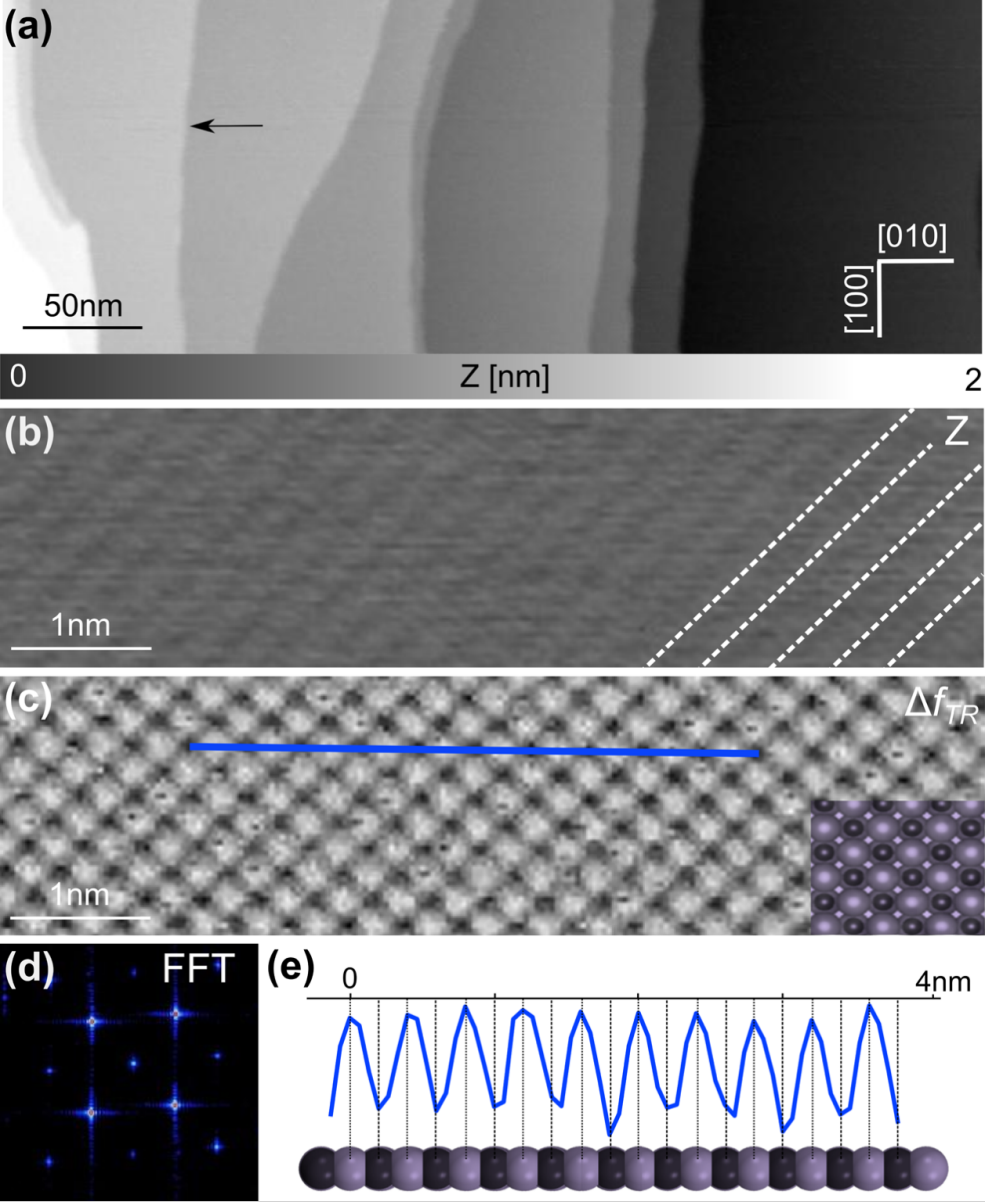
From large-scale imaging to atomic resolution on NiO(001). (a) Large scale topographic image of the NiO(001) crystal presenting clean terraces (scan parameters: 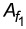 = 4 nm, Δ*f*_1_ = −30 Hz). The high-resolution topographic image (b) shows the atomic rows while the torsional frequency shift (c) indicates that only one kind of atom can be imaged: one element appears as bright protrusion, the other as darker hole (scan parameters: 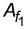 = 7 nm, 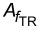 = 80 pm, Δ*f*_1_ = −79 Hz). (d) Fast Fourier transform (FFT) of (c) shows the expected cubic packing of the crystal. (e) Profile acquired along the dark blue line visible in (c). The spacing between the atoms (410 pm) corresponds well to the theoretical bulk lattice constant of NiO(001): *a* = 417 pm.

Figure 2 shows topography images of the bare NiO(001) surface measured by bimodal nc-AFM using both the first normal and torsional resonance frequency of the cantilever. In this mode high stability and resolution can be combined in order to get detailed information at the atomic scale even under room-temperature conditions [46]. The large-scale measurement shown in Figure 2a presents large terraces reaching more than 100 nm in width. Mono-atomic steps, which are 210 ± 10 pm high, can be identified as indicated by the black arrow.

Moreover, atomic-scale information of the surface was obtained: The atomic rows along the 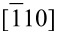 crystallographic direction were resolved using the first oscillation mode (*f*_1_), whereas single atoms could be seen in the simultaneously recorded frequency shift signal of the torsional resonance mode (Δ*f*_TR_) (Figure 2b,c). The data were acquired while regulating the tip–sample distance using a constant frequency shift Δ*f*_1_ [47–48]. Moreover, the Fast Fourier transform (Figure 2d) of Figure 2c, exhibits a cubic faced-centred lattice with a mesh parameter of 415 pm consistent with the theoretical bulk lattice constant of the NiO(001) surface (*a* = 417 pm). Figure 2e presents the profile recorded along the blue line visible in Figure 2c and demonstrates that each protrusion corresponds to every second atom.

Thus, similar to what is observed for other materials [49–50], only one type of atom, either Ni or O, is visible in our nc-AFM measurements. Since no atomic defects have been observed nor any contrast inversion, as for example in measurements on TiO_2_ [51], and considering that Ni vacancies are supposed to occur more frequently in NiO [52], it can be assumed that the protrusions are related to oxygen atoms. This is also in agreement with earlier experimental studies on NiO where bright protrusions have been assigned to O atoms when imaged with a metallic tip [35–37,39,41]. This interpretation is supported by calculations for a quite similar metal/metal-oxide system [53], or for the image contrast in AFM measurements on the NiO(001) surface, predicting a stronger interaction of metal tips with the oxygen atoms [54]. Nevertheless, the latter argument has to be treated carefully since it is well known that metallic tips exhibit a stronger interaction with surface anions on polar surfaces [55].

### DCPDMbpy adsorbed on NiO(001)

Knowing the atomic structure of the clean NiO(001) surface, we investigated the adsorption properties of the anchoring ligand DCPDMbpy, at various coverages. Depending on the deposition conditions and post-deposition treatments of the sample, DCPDMbpy molecules remain separated or form molecular clusters or islands on the surface of NiO(001) at room temperature.

**Figure 3:**
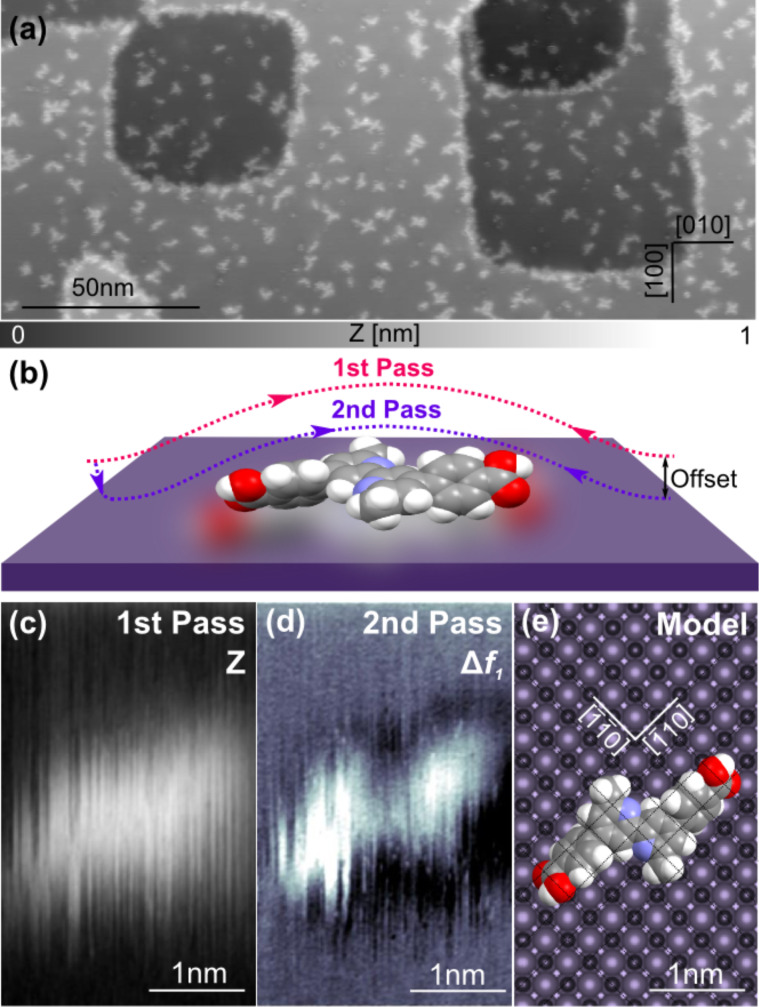
Single DCPDMbpy molecules on NiO(001). (a) Topographic image of NiO(001) covered with DCPDMbpy after low-coverage exposure (scan parameters: 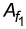 = 4 nm, Δ*f*_1_ = −3 Hz). (b) Sketch of the multipass technique. (c) Topographic image of a single DCPDMbpy molecule adsorbed an a terrace acquired in the first scanning pass (scan parameters: 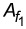 = 4 nm, Δ*f*_1_ = −2.5 Hz). (d) Frequency shift image recorded in the second scanning pass with an offset of −350 pm showing that DCPDMbpy on the surface is in the trans-conformation. (e) Model of DCPDMbpy in a trans-conformation on the NiO(001) surface.

Figure 3a presents a large-scale topographic image of the surface after a low-coverage deposition process (0.2 monolayers) without any post-annealing treatment. The sample exhibits large NiO(001) terraces separated by monoatomic steps and covered with DCPDMbpy molecules. The different sizes and shapes of the protrusions indicate that both single molecules and molecular clusters can be identified under these conditions. DCPDMbpy exhibits a clear tendency to adsorb preferentially at step edges, both at the upper and lower sides. This propensity to decorate the step edges has already been observed for several molecules on ionic crystals [56–57]. Comparably, it has been observed on metal oxides that molecular islands can arise undergoing a step-flow growth process [41,58]. However, depending on the studied molecule, it has also been shown that some are not adsorbed preferentially at the step edges but are homogeneously distributed all over the surface of the same oxides [8,59] suggesting a strong interaction with the substrate. In the present case, the interval in-between the single molecules or clusters (3.9 ± 0.7 nm in average, corresponding to a relative distance of about twice the length of a DCPDMbpy molecule) on the terraces indicates that the diffusion is limited, implying also a relatively strong adhesion to the surface at specific sites. To reduce the binding energy of the molecules and stimulate a self-assembly, the sample was annealed for 1 h at 150 °C. However, no enhanced diffusion or island formation of the organic ligand at this low surface coverage and under these annealing conditions was observed (see Figure S1 in Supporting Information File 1). Also annealing at higher temperatures did not increase the mobility of the molecules, but rather led to their desorption.

**Figure 4:**
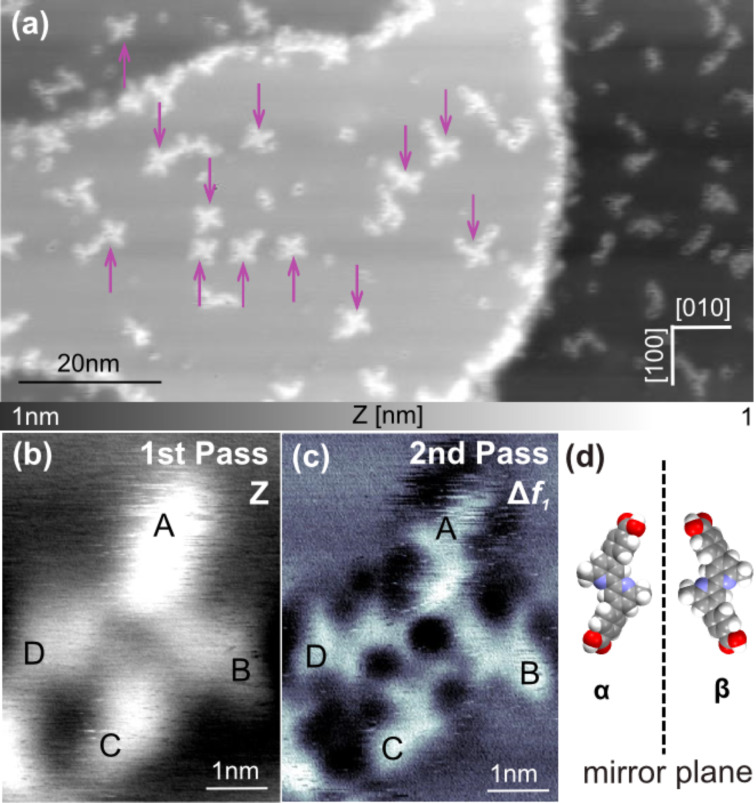
Windmill-shaped cluster on NiO(001). (a) Topographic image of DCPDMbpy forming molecular clusters on NiO(001). Violet arrows show that DCPDMbpy often form windmill-shaped clusters. (b) Topographic image of a molecular cluster formed by four DCPDMbpy molecules acquired in the first scanning pass (scan parameters: 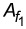 = 4 nm, Δ*f*_1_ = −2.8 Hz). (c) Frequency shift image recorded in the second scanning pass with an offset of −350 pm showing that DCPDMbpy can exhibit different conformations. (d) Sketch of the two different surface enantiomers of trans-DCPDMbpy.

The adsorption geometry of DCPDMbpy on a NiO(001) terrace was resolved by using the multipass technique to reach submolecular resolution without a functionalized tip. This technique was recently introduced by Moreno et al. for low temperatures [60]. In principle, this method consists of recording a first scan line with a closed feedback loop where the tip–sample distance is regulated using a topographic set point Δ*f*_1_ and then acquiring a second scan in a open feedback loop following the recorded topography but applying an additional constant *Z*-offset, reducing the tip–sample distance (Figure 3b). Here, we show, that this technique can also be used at room temperature to gain detailed information at submolecular scale. Due to unstable scanning conditions over step edges and large corrugations of the surface at these specific spots, the multipass technique was successfully applied only for molecules adsorbed on terraces. Figure 3c shows the topographic image of DCPDMbpy on a NiO(001) surface acquired during the first scan using this method. The size is consistent with the molecular dimensions, (i.e., length ≈ 1.8 nm and height ≈ 150 pm) and suggests that a single molecule is lying flat on the surface with phenyl and pyridine rings slightly twisted with respect to each other to minimize H–H repulsions. The adsorption geometry in a *trans*-conformation is revealed in the Δ*f*_1_ image of the second pass where a *Z*-offset of −350 pm towards the surface was applied (Figure 3d). Based on the known atomic structure of the specific surface, resolved in Figure 2c, a model of the adsorption geometry of DCPDMbpy is displayed in Figure 3e. It shows that DCPDMbpy adsorbs according to the atomic symmetry of the substrate and aligns along the 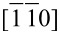 and 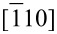 crystallographic directions of the surface in such a way that the two similar functional groups are always facing the same type of surface atom (either Ni or O). For instance, taking into account the partial charge distributions of the surface (Ni is δ^+^) and of the bpy unit (N is δ^−^), we might expect a positioning of the nitrogen atoms (in blue in the Figure) on top of the nickel atoms. However, even at low coverage, single molecules are less regularly observed than molecular clusters. Indeed, as shown in Figure 4a by the arrows, DCPDMbpy appears frequently to aggregate in windmill-shaped clusters.

A preferential orientation along the atomic rows of the substrate for this particular type of cluster is visible as observed for single molecules (see Figure 3e). Each DCPDMbpy in a cluster appears to lie flat implying that the adsorption geometry of the molecule on NiO(001) is the same when adsorbing as a single molecule or when forming clusters. One of the windmill-shaped clusters is resolved in Figure 4b,c. The topographic image (Figure 4b) shows clearly that the cluster is composed of four distinct molecules. Figure 4c, which was recorded during the second pass with a *Z*-offset of −350 pm, presents the same cluster with intramolecular contrast. DCPDMbpy molecules are adsorbed in a *trans*-conformation and molecules A, B and C are rotated by 90° clockwise with respect to each other in contrast to molecule D the orientation of which cannot be obtained by any rotation of the other molecules. Therefore, DCPDMbpy presents two distinct chiralities. In fact, like many other molecules, DCPDMbpy is prochiral, when confined in two dimensions [61–65], implying the appearance of the surface enantiomers: α-DCPDMbpy and β-DCPDMbp (Figure 4d). In the case of the specific cluster imaged in Figure 4c it can be seen that the molecules A, B and C are in α-conformation whereas molecule D is the β-form of DCPDMbpy. The ratio α/β varies from one cluster to the other, suggesting that there is no preferred conformation.

**Figure 5:**
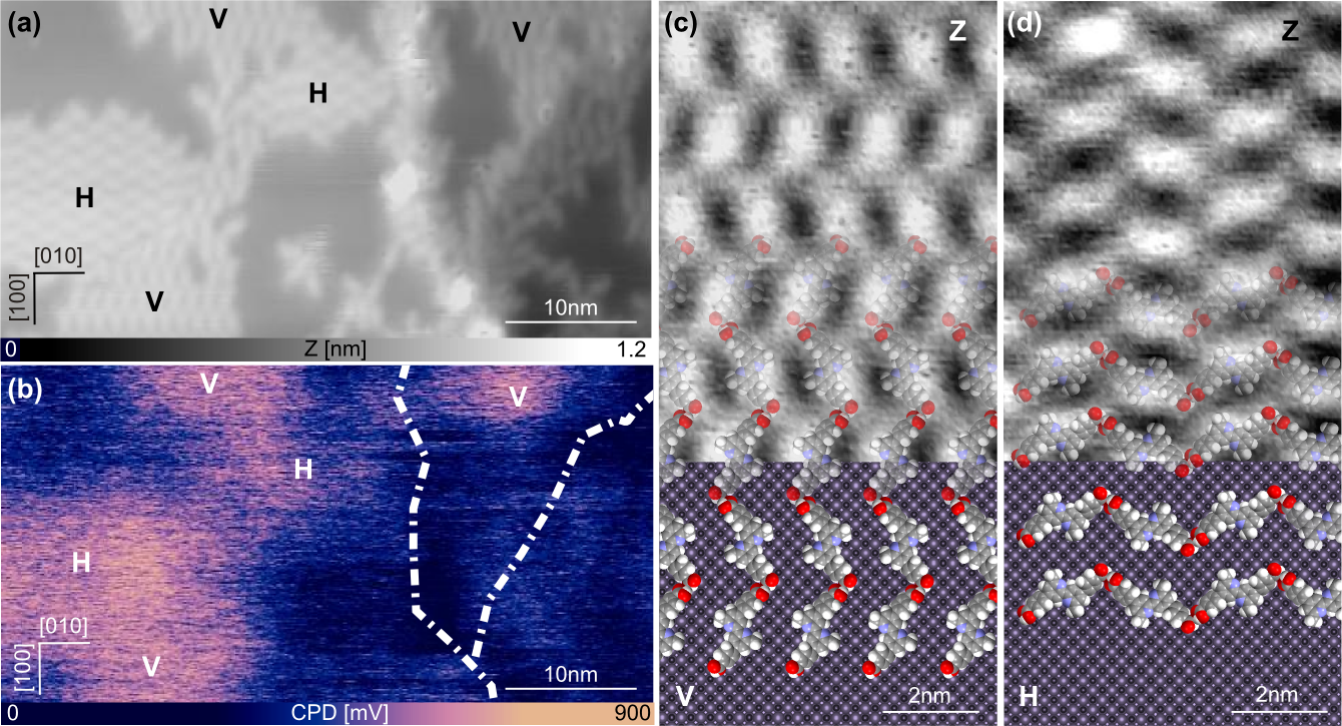
Islands of DCPDMbpy on NiO(001). (a, b) simultaneous topographic and CPD measurements of the molecular islands (V and H) on the NiO(001) substrate, respectively. (c, d) Close-up on both types of islands. In the lower parts qualitative models of the substrate and the molecular arrangement are overlayed. (Scan parameters: 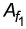 = 4 nm; Δ*f*_1_ = −9 Hz; *f*_ac_ = 900 Hz; *V*_ac_ = 800 mV.)

With an increased coverage (0.7 monolayers) followed by a post-annealing process (1 h at 150 °C) molecular islands were formed. Figure 5a shows that the step edges are completely saturated with molecules whereas terraces are covered with organized islands. Interestingly, it can be seen that the islands avoid growing from step edges contrary to what is often observed at room temperature [58,66–67]. This implies that adsorption at the step edges is probably different to the terraces. Two orientations of the islands are visible in Figure 5a; in the first (orientation V), the DCPDMbpy molecules form vertical rows following the [100] direction of the NiO surface while they align in horizontal rows extended in the [010] direction in the second (orientation H). The heights of 180 ± 20 pm of both arrays suggest that the molecules are still lying flat on the surface in the same configuration as described above, independently of the island orientation.

Figure 5b shows the contact potential difference (CPD) simultaneously recorded by Kelvin probe force microscopy (KPFM) [68]. The CPD arises from the work function difference between the tip and the substrate and is altered by surface charges or dipoles. The voltage needed to compensate for the electrostatic forces due to this potential difference is measured in KPFM. The CPD image (Figure 5b) shows that this voltage is the same for islands with V and H orientations, indicating that the adsorption geometry of DCPDMbpy, influencing either the charge transfer between the surface and the molecules or the intramolecular dipole strength and orientation, is identical. The absolute CPD is increased above the islands compared to the bare NiO surface. In areas with a lower molecule density, e.g., at step edges and certain areas on the terraces, the CPD contrast is much weaker implying a direct influence of the self-assembly on the electrical surface properties. In summary, compared to bare NiO, the CPD is increased when molecules are adsorbed in assemblies and consequently, the sample work function also increased. This can directly be related to a more negatively charged molecular layer compared to the substrate and a dipole moment is pointing towards the surface.

The average CPD difference between the islands and the surface is measured to be Δ*V*_CPD_ ≈ 180 ± 30 mV (see Figure S2 in Supporting Information File 1). Knowing that the difference in dipole moment densities (Δ*p*) can be calculated by the following formula [69–70]:


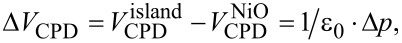


the dipole moment density difference can be extracted to be 0.5 D·nm^−2^. Taking into account a measured molecule density of 0.43 nm^−2^, which could be calculated thanks to high-resolution imaging, an average dipole moment of 1.1 D per molecule is estimated. This dipole moment is directed towards the NiO surface and can be attributed to a partial charge transfer from the surface to the molecules in average of 0.3 electrons per molecule if assuming an effective surface molecule distance of 90 pm, corresponding to half of the measured island height.

Figure 5c,d shows a close-up of the two different orientations, V and H, respectively. In both cases it can be seen that the molecules are arranged with alternating orientations suggesting that every second molecule exhibits a different conformation (α-DCPDMbpy or β-DCPDMbpy), confirming that the ratio α/β on the NiO surface is 1:1. This image also clearly shows that orientation V can be obtained by rotating orientation H by 90°, and vice versa, which is consistent with the symmetries of the NiO(001) surface. In the lower part of Figure 5c and Figure 5d, a qualitative model based on the dimensions and orientations of the molecules is superimposed on the measurements. This model additionally suggests that the molecules are linked to each other via H-bridging of their carboxylic groups. This interaction, however, needs an activation induced by increasing the molecule density on the surface. Furthermore, this arrangement allows for electrical coupling and, consequently, also for charge transfer between the NiO surface and the dye precursor molecule DCPDMbpy.

## Conclusion

We have presented high-resolution topographic measurements using bimodal nc-AFM at room temperature of the anchoring part of a larger dye molecule (DCPDMbpy) adsorbed on a NiO(001) crystal surface. The surface structure of NiO(001) was resolved with atomic resolution using the first resonance and the torsional resonance. Depending on the deposition rate, single molecules, molecular clusters, and molecular islands have been imaged. Through the so-called multipass technique, submolecular resolution could be achieved and direct evidence of flat-lying molecules on the substrate with *trans*-conformation could be demonstrated. Furthermore, DCPDMbpy exhibits a chiral character upon confinement on a surface leading to the appearance of two different surface enantiomers. Upon increasing the coverage, molecular islands with two symmetric orientations (V and H) appeared based on flat-lying molecules with alternating enantiomeric form. These islands exhibit the particular disposition not to grow starting from step edges, suggesting that the adsorption geometry of DCPDMbpy is probably different at those sites. Nevertheless, we showed, through qualitative models, that the adsorption symmetries of DCPDMbpy with respect to the crystallographic direction on the (001) terraces of NiO are the same, regardless whether they are single, forming windmill clusters or packing in islands. By combined nc-AFM and KPFM measurements an average charge transfer from the NiO surface to molecules in the islands of 0.3 electrons per molecule was determined.

## Experimental

### Synthesis

DCPDMbpy was synthesized by Ana Hernández Redondo (University of Basel) following the reported procedure [71].

### Sample preparation

The NiO(001) crystals used in this study were purchased from SurfaceNet. They consist of a rectangular rod with dimensions 2 × 2 × 7 mm^3^ and a long axis in the [001] direction. The NiO(001) surface was prepared trough in situ cleavage with prior and subsequent annealing (at 600 °C and 500 °C, respectively) resulting in an atomically clean surface. A powder of DCPDMbpy molecules was thermally evaporated at 255 °C on the NiO surface kept at room temperature under ultra-high vacuum (UHV) conditions (*p <* 1 × 10^−10^ mbar) with a deposition rate of 0.5 Å/min. The deposition time was fixed between 10 s and 2 min depending on the desired coverage. The samples were then annealed 1 h at 150 °C to facilitate the diffusion of the molecules on the substrate.

### Scanning probe microscopy

The measurements were carried out with a custom-built atomic force microscope (AFM) in UHV and at room temperature. All AFM images were recorded in the non-contact mode (nc-AFM), using silicon cantilever (Nanosensors PPP-NCR, stiffness *k* = 20–30 N/m, resonance frequency *f*_1_ around 165 kHz, *Q_f_*_1_ factor around 30000, torsional frequency *f*_TR_ around 1.5 MHz, and *Q*_TR_ factors around 100000) with compensated contact potential difference (CPD). Kelvin probe force microscopy was performed in frequency-modulation mode using an electrical oscillation at a frequency of *f*_ac_ = 900 Hz and with an amplitude *V*_ac_ = 800 mV applied together with the DC compensation voltage to the sample.

## Supporting Information

File 1 Additional experimental data.
Supporting Information shows the influence of the temperature on the diffusion of the molecules and discusses the determination method of the average CPD difference.
