# Supplementary material for: Anchoring of a dye precursor on NiO(001) studied by non-contact atomic force microscopy
Source: Beilstein J Nanotechnol. 2018 Jan 23;9:242–9. doi: 10.3762/bjnano.9.26 (PMC5789385; doi:10.3762/bjnano.9.26)
Supplement: File 1 — Additional experimental data. Supporting Information shows the influence of the temperature on the diffusion of the molecules and discusses the determination method of the average CPD difference. [file Beilstein_J_Nanotechnol-09-242-s001.pdf]

Supporting Information

for

Anchoring of a dye precursor on NiO(001) studied by non-contact atomic force microscopy

Sara Freund[1], Antoine Hinaut[1], Nathalie Marinakis[2], Edwin C. Constable[2], Ernst Meyer[1], Catherine E. Housecroft[2] and Thilo Glatzel[*1]

Address: [1]Department of Physics, University of Basel, Klingelbergstrasse 82, 4056 Basel, Switzerland and [2]Department of Chemistry, University of Basel, BPR 1096, Mattenstrasse 24a, 4058 Basel, Switzerland

Email: Thilo Glatzel - thilo.glatzel@unibas.ch

* Corresponding author

Additional experimental data

Temperature influence

Figure S1 illustrates the influence of annealing on DCPDMbpy adsorbed on NiO(001) for two different coverages. At low coverage (10 s exposure time with a deposition rate of 0.5 Å/min, which leads approximatively to a coverage of 0.2 monolayers), it can be seen in Figure S1a that most of the molecules are forming clusters, and show a tendency to adsorb at step edges. A small average distance between the molecules (3.9 ± 0.7 pm) and the fact that the step edges are not completely covered demonstrate that the diffusion rate of DCPDMbpy on NiO(100) is pretty low.

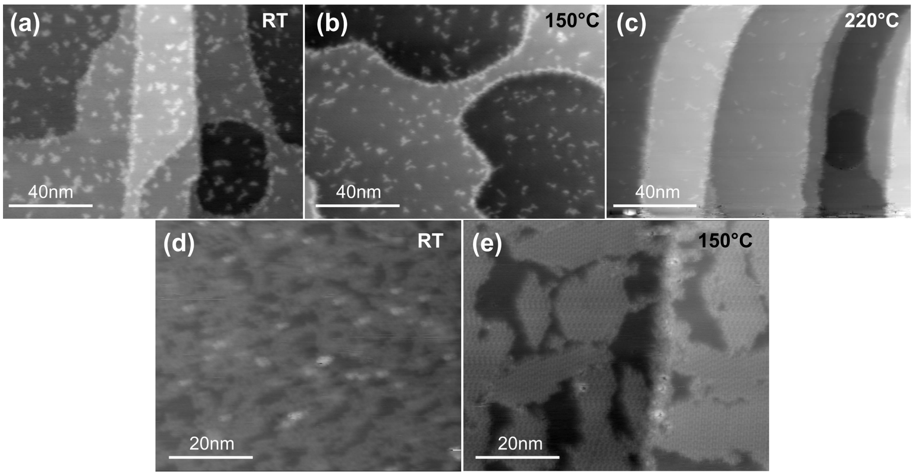

Figure S1: Temperature influence. (a–c) DCPDMbpy adsorbed at RT on NiO(001) under low-coverage conditions, before and after 1 h of annealing at a temperature of 150 °C and then 220 °C respectively. (d–e) DCPDMbpy adsorbed at RT on NiO(001) under high-coverage conditions, before and after 1 h of annealing at 150 °C, respectively. Scan parameters: A_{f_1} = 4 nm; $\Delta f_1 = -5.1$ Hz $\Delta f_1 = -7$ Hz, $\Delta f_1 = -9$ Hz and $\Delta f_1 = -18$ Hz, respectively.

After annealing at 150 °C for 1 h, it can be seen in Figure S1b that this diffusion rate can be slightly increased, since the average distance between the clusters is a little larger (5.3 ± 0.5 pm) and the step edges are completely saturated with molecules. Nevertheless, no drastic enhancement of the diffusion nor island formation was observed. Annealing at higher temperatures (220 °C for 1 h) does not increase the mobility of the molecules, but rather leads to their desorption. Indeed it can be seen in Figure S1c that the molecular density decreased drastically and that the step edges are less decorated.

At higher coverage (2 min exposure time with a deposition rate of 0.5 Å/min, which leads approximatively to a coverage of 0.7 monolayers), annealing at 150 °C for 1 h influences clearly

the morphology of the interface. Before annealing, the surface of bare NiO is barely visible and almost completely covered with randomly packed molecules (Figure S1d). This suggests again that the diffusion of DCPDMbpy is really low at RT but increases at higher temperatures. Indeed, after the annealing process molecules are forming crystalline islands (Figure S1e). Island formation is only possible at high coverage, since it was shown that the diffusion rate cannot be drastically increased. Molecules have to be already really close to each other to reorganize in islands.

Average CPD difference determination

Average CPD difference ($\Delta_{CPD} = CPD_{island} - CPD_{NiO}$) was measured as follows: Two different masks were drawn on the topographic nc-AFM images. In Figure S2a and Figure S2b, a first mask (in red) was superimposed to the islands and a second mask (in blue) was superimposed to the bare NiO surface. Steps and island edges were excluded from the measurements to avoid edge effects that can strongly influence the CPD. These two masks were then distributed to the corresponding KPFM images Figure S2c,d and the average CPD was measured in these areas. This operation was repeated on a set of several images to reduce the margin of error. The CPDs were slightly changing from one image to another since their values are strongly tip-dependent. Nevertheless, the CPD difference was constant and determined to be: $\Delta_{CPD} = 180 \pm 30$ mV.

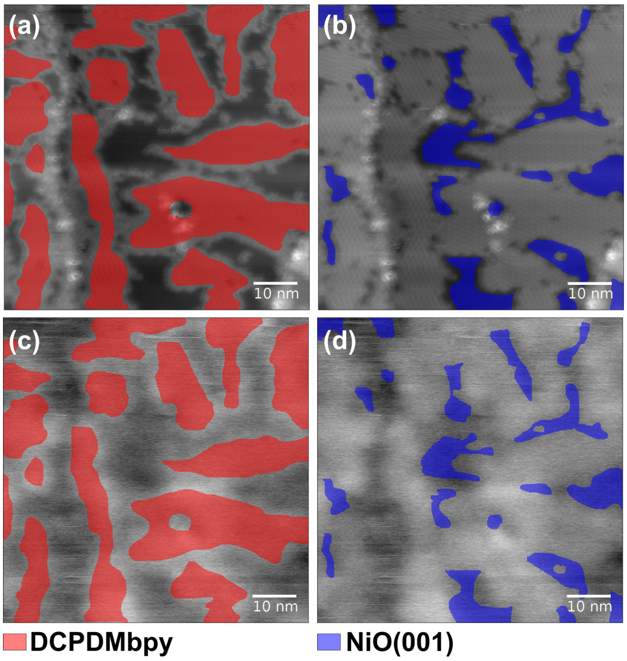

Figure S2: (a,b) nc-AFM topographic images superimposed with a mask on molecular islands (red) and on the bare NiO substrate (blue), respectively. (c,d) KPFM images superimposed with corresponding masks. Scan parameters: $A_{f_1} = 4$ nm; $\Delta f_1 = -11.5$ Hz; $f_{AC} = 900$ Hz; $V_{AC} = 800$ mV.
